# The mechanisms through which the family music environment influences young children’s empathy: the mediating role of music emotional contagion and the moderating role of parent–child relationship quality

**DOI:** 10.3389/fpsyg.2026.1788616

**Published:** 2026-03-19

**Authors:** Yuanyuan Zhou

**Affiliations:** College of Music and Dance, Huaqiao University, Xiamen, Fujian, China

**Keywords:** children’s empathy, family music environment, moderated mediation model, music emotional contagion, parent–child relationship quality

## Abstract

**Objective:**

To explore the psychological mechanisms underlying the association between the family music environment and young children’s empathy, with a specific focus on the mediating role of music emotional contagion and the moderating role of parent–child relationship quality.

**Methods:**

A quantitative research design combining cross-sectional surveys and situational experiments was employed. A total of 368 parent–child dyads (children aged 3–6 years) were recruited from six public kindergartens across eastern, central, and western China. Measurements included the revised Family Music Environment Scale (FMES), Young Children’s Empathy Rating Scale (YCERS), Music Emotional Contagion Scale (MECS), and Parent–Child Attachment Scale (PCAS). Data were analyzed using SPSS 26.0, AMOS 24.0, and Hayes’ PROCESS macro.

**Results:**

(1) The family music environment, music emotional contagion, parent–child relationship quality, and children’s empathy were all significantly and positively correlated (*p* < 0.01). (2) Statistical analysis indicated that music emotional contagion mediated the relationship between the family music environment and children’s empathy (indirect effect = 0.185, 95% CI [0.138, 0.239]). (3) Parent–child relationship quality did not significantly moderate the path from family music environment to music emotional contagion (interaction *p* > 0.05), although it exerted a significant main effect on music emotional contagion ( β = 0.295, *p* < 0.001).

**Conclusion:**

The family music environment appears to promote children’s empathy not directly, but largely by activating the mechanism of music emotional contagion. This pathway remained robust across varying levels of parent–child relationship quality, suggesting that the family music environment may serve as a potential compensatory resource for fostering socio-emotional development in early childhood.

## 1 Introduction

Empathy, defined as the core socio-emotional capacity to perceive, understand, and respond to the emotional states of others, serves as a cornerstone for social development in early childhood. It is closely associated with the quality of peer interactions, the cultivation of prosocial behaviors, and long-term psychological well-being (Spinrad and Eisenberg, 2019). The Learning and Development Guide for Children Aged 3–6 in China explicitly identifies “caring for and respecting others” and “noticing others’ emotions and expressing concern” as central objectives for interpersonal development, highlighting the foundational role of empathy cultivation in preschool education.

From the perspective of Ecological Systems Theory (Bronfenbrenner, 1981), the family constitutes the central microsystem for child development. Within this system, interaction patterns, cultural atmosphere, and activity experiences play a significant role in shaping children’s emotional perception and social adaptation through bidirectional processes. As a vital component of the family’s cultural ecology, the family music environment—characterized by its unique capacity for emotional arousal and affective transmission—is increasingly recognized as a key variable influencing early socio-emotional development (Politimou et al., 2018). In contemporary society, where deficits in social competence affect a significant proportion of young children, exploring pathways for nurturing empathy within the family context possesses both urgent practical significance and theoretical value.

Existing literature has provided preliminary evidence linking the family music environment to child development. For instance, classic studies have shown that joint music-making significantly promotes prosocial behavior in young children (Gerry et al., 2012; Kirschner and Tomasello, 2010) and that interpersonal synchrony during musical activities strengthens emotional bonding (Cirelli et al., 2014; Williams et al., 2015). More recent research continues to support these findings, suggesting that musical engagement is intrinsically linked to socio-emotional competencies. For example, Savage et al. (2020) highlighted music’s evolutionary function in fostering social bonding across cultures, while Cuervo and Campayo (2024) further demonstrated that musical interaction can specifically target and enhance the cognitive and affective components of empathy in educational settings. Despite these advances, three notable gaps remain, underscoring the necessity of the present study. First, the research focus has been somewhat generalized; existing studies largely concentrate on the macroscopic effects of music on cognitive development or general prosocial behavior, and have yet to fully elucidate the mechanisms acting upon empathy as a distinct construct. Second, the investigation of mediating mechanisms lacks precision. Prior research has often relied on broad variables such as emotional regulation, overlooking the core attribute of music itself: emotional contagion. Music acts as a unique vector for emotional transmission; the process of emotional resonance it triggers (i.e., music emotional contagion) is proposed to represent a critical “bridge” connecting environmental input to empathetic capacity, yet this mechanism remains under-verified in the context of family environments. Third, the role of moderating variables, specifically the parent–child relationship, is insufficiently understood. While high-quality parent–child relationships are known to enhance the positive effects of the family environment, it remains unclear whether the parent–child relationship moderates the specific impact of the family music environment on empathy. Specifically, does the efficacy of music in fostering empathy vary depending on the quality of parent–child attachment?

Furthermore, existing research often lacks theoretical integration, failing to systematically synthesize Emotional Contagion Theory, Attachment Theory, and Ecological Systems Theory into a unified “Environment-Process-Development-Boundary” framework. To address these gaps, this study constructs an integrated theoretical framework. First, Ecological Systems Theory provides the overarching logic. It posits that interactive experiences within the family microsystem provide the context for development. The family music environment, as a carrier of culture and activity, creates emotional exchange contexts through parent–child musical interactions, constituting key external stimuli for empathy development. The parent–child relationship, as the core interactive dimension, serves as a boundary condition that may regulate the intensity and direction of these external stimuli.

Second, we integrate Emotional Contagion Theory and the “Emotional Resonance Theory” from music psychology to underpin the mediating role of music emotional contagion. Hatfield et al. (1994) described emotional contagion as a process of “perception-mimicry-synchrony.” As a non-verbal emotional symbol system, music’s melody, rhythm, and harmony can directly trigger emotional perception (Juslin and Laukka, 2003). For young children, whose cognitive and emotional processing is highly situated, joint listening and musical play allow them to perceive musical emotions while simultaneously observing and mimicking parental emotional expressions. Through a chain of “musical emotional activation–parent-child emotional synchronization–internalization of emotional experience,” children may gradually acquire the capacity for emotional recognition and empathetic response. Furthermore, research suggests that music emotional contagion has cross-situational universality and is amplified in parent–child contexts due to emotional bonding (Trainor and Cirelli, 2015), supporting its potential role as a mediator.

Third, we draw on Attachment Theory’s “Internal Working Models” and Family Systems Theory to clarify the potential moderating role of the parent–child relationship. Jones and Bowlby (1970) posited that attachment quality shapes internal working models of emotion. Children with secure attachment may form positive cognitions that emotions are shareable, facilitating the internalization of empathy during musical interactions. Conversely, insecure attachment may lead to defensive processing, potentially weakening the mediation effect (Ainsworth et al., 1978). Concurrently, Family Systems Theory suggests that the functional state of the parent–child subsystem influences the efficacy of other environmental elements. High-quality relationships may optimize “emotional transmission efficiency,” whereas low-quality relationships could create barriers.

Accordingly, this study aims to systematically examine the mechanisms through which the family music environment is associated with young children’s empathy, specifically testing the mediating effect of music emotional contagion and the moderating effect of parent–child relationship quality. Theoretically, this study seeks to fill gaps regarding direct associations, shed light on the “black box” of psychological mechanisms via emotional contagion, and explore boundary conditions. Practically, the findings intend to provide evidence-based strategies for parents to optimize family music environments, offering a low-threshold pathway to foster children’s socio-emotional health.

## 2 Participants and methods

### 2.1 Participants

The present study employed a stratified random sampling strategy to recruit participants from six public kindergartens located in eastern, central, and western China. To ensure a representative sample of socioeconomic backgrounds, the selection included four urban kindergartens and two township kindergartens. The target population consisted of preschool children aged 3 to 6 years and their primary caregivers. An *a priori* power analysis was conducted using G*Power 3.1.9.7 (Faul et al., 2007) to determine the necessary sample size. Based on a moderated mediation analytical framework, the parameters were set with a medium effect size ( $f^{2}=0.15$ ), a significance level ( α ) of 0.05, and a statistical power ( 1−β ) of 0.95. The calculation indicated that a minimum of 384 parent–child dyads was required to detect statistically significant effects.

Following the initial recruitment, a total of 368 valid questionnaires were obtained, resulting in an effective response rate of 95.8%. Although the final sample size fell slightly short of the theoretical target, a *post-hoc* power analysis confirmed that the statistical power for the obtained sample (*N* = 368) exceeded 0.90, which is well above the conventional threshold of 0.80, thereby ensuring the robustness of the statistical tests.

The demographic composition of the children included 192 boys (52.2%) and 176 girls (47.8%). Regarding age distribution, the sample comprised 92 three-year-olds (25.0%), 115 four-year-olds (31.3%), 103 five-year-olds (28.0%), and 58 six-year-olds (15.7%). In terms of parental educational attainment, 105 parents (28.5%) held a junior college degree or below, 213 (57.9%) held a bachelor’s degree, and 50 (13.6%) possessed a master’s degree or higher.

Ethical approval for this study was granted by the Ethics Review Committee of Huaqiao University. Prior to data collection, written informed consent was obtained from all participating parents and kindergarten administrators, ensuring that all participants were fully aware of the study’s purpose and their rights.

### 2.2 Measures

1. Family Music Environment Scale (FMES)

The home musical climate was assessed using the revised Family Music Environment Scale (FMES). This instrument was adapted from the Music@Home questionnaire originally developed by Politimou et al. (2018) and was culturally validated for use in the Chinese context. The scale consists of 22 items distributed across three dimensions: Music Resource Provision, Parent–Child Music Interaction, and Transmission of Musical Attitudes. Participants rated items on a 5-point Likert scale ranging from 1 (*strongly disagree*) to 5 (*strongly agree*), with higher aggregate scores indicating a richer family music environment. In the current study, the scale demonstrated excellent internal consistency, with a Cronbach’s α coefficient of 0.91. Furthermore, Confirmatory Factor Analysis (CFA) yielded satisfactory fit indices ( $\chi^{2}/\mathrm{df}=2.36$ , *CFI* = 0.95, *RMSEA* = 0.06) based on established criteria (Hu and Bentler, 1999), supporting the construct validity of the measure.

1. Young Children’s Empathy Rating Scale (YCERS)

To evaluate children’s empathy, we utilized the Young Children’s Empathy Rating Scale (YCERS), which was developed based on Eisenberg et al. (2013) theoretical framework of empathy development and adapted from measurement tools by Dadds et al. (2008). The scale comprises 18 items assessing three distinct dimensions: Emotion Recognition, Emotion Understanding, and Empathetic Response. To mitigate the potential bias inherent in single-source reporting, empathy scores were derived from a composite of ratings provided by both parents and head teachers, with each source contributing an equal weight of 50%. Psychometric analysis for this study indicated high reliability and validity, with a Composite Reliability (CR) of 0.92 and an Average Variance Extracted (AVE) of 0.71. The measurement model showed good fit ( $\chi^{2}/\mathrm{df}=2.14$ , *CFI* = 0.96, *RMSEA* = 0.05).

1. Music Emotional Contagion Scale (Child Version) (MECS)

Assessing emotional contagion in young children requires an age-appropriate approach; thus, an adapted child version of the Music Emotional Contagion Scale was employed. Grounded in Juslin et al. (2015) BRECVEMA model and the foundational work of Doherty (1997), this instrument contains 12 items that measure Emotional Arousal, Emotional Resonance, and Emotional Transmission. Considering the cognitive limitations of preschool children, a situational experiment method was implemented. Children were exposed to three standardized musical excerpts designed to evoke happy, sad, and calm emotions, respectively. Following each excerpt, children identified their induced emotional state using visual aids (facial expression cards). The scale demonstrated good internal consistency in the present sample (Cronbach’s α = 0.88).

1. Parent–Child Relationship Quality Scale (PCAS)

The quality of the parent–child relationship was measured using the Chinese version of the Parent–Child Attachment Scale (PCAS), which is rooted in Ainsworth et al.’s (1978) attachment theory. For the purpose of this study, the Secure Attachment dimension was selected as the primary indicator. This dimension consists of 20 items, with higher scores reflecting a more secure and harmonious parent–child bond. The scale exhibited strong reliability in the current sample, as evidenced by a Cronbach’s α of 0.89.

### 2.3 Procedure

The study followed a quantitative research design implemented in three sequential stages. The initial preparation stage involved the rigorous training of 12 experimenters to standardize the administration of surveys and situational experiments. Only experimenters who passed a competency assessment were permitted to conduct fieldwork.

During the data collection stage, a multi-faceted approach was used. Parents completed the FMES, PCAS, and demographic questionnaires via a secure online platform, which included built-in logic checks to minimize missing data and ensure response validity. Concurrently, the situational experiment for music emotional contagion was conducted in a quiet, dedicated room within each kindergarten. To ensure consistency, experimenters played the standardized musical excerpts and guided children through the MECS using the visual aids. Additionally, classroom teachers independently completed the relevant sections of the YCERS for each child in their class.

Finally, the data processing stage involved matching data from parent, teacher, and experimenter sources. Data were screened for outliers and missing values. Cases with less than 10% missing data were handled using multiple imputation techniques to maximize the retention of valid information.

### 2.4 Statistical analysis

All statistical analyses were performed using SPSS version 26.0 and AMOS version 24.0. First, to address the potential issue of common method bias arising from self-reported measures, Harman’s single-factor test was conducted. Second, descriptive statistics (means and standard deviations) and Pearson correlation coefficients were computed to examine the preliminary relationships among the family music environment, music emotional contagion, parent–child relationship quality, and children’s empathy. Finally, the proposed hypotheses were tested using Hayes (2013) PROCESS macro. Specifically, Model 4 was utilized to test the mediation effect of music emotional contagion, while Model 7 was employed to examine the moderated mediation effect involving parent–child relationship quality. To ensure robust inference, bias-corrected bootstrapping with 5,000 resamples was applied (Preacher and Hayes, 2008) to estimate 95% confidence intervals (CIs), with statistical significance defined as *p* < 0.05.

## 3 Results

### 3.1 Assessment of common method bias

Given that the data regarding the family music environment, parent–child relationship quality, and children’s empathy were partially derived from parent self-reports, there is a potential risk of common method bias (CMB). To address this, Harman’s single-factor test was conducted by entering all measurement items into an unrotated exploratory factor analysis. The results revealed multiple factors with eigenvalues greater than 1. However, the first factor accounted for 56.78% of the total variance. This value exceeds the commonly recommended threshold of 40% (Podsakoff et al., 2003), suggesting the presence of common method variance in the dataset. While the Confirmatory Factor Analysis (CFA) reported in the Methods section demonstrated satisfactory model fit indices ( $\chi^{2}/\mathrm{df}<3$ , *CFI* > 0.90, *RMSEA* < 0.08) supporting the discriminant validity of the constructs, we explicitly acknowledge that the shared method variance represents a limitation that may inflate the observed correlations. This potential bias is further addressed in the Discussion section.

### 3.2 Descriptive statistics and correlation analysis

The means, standard deviations, and Pearson correlation coefficients for all study variables are presented in Table 1. The descriptive results indicate that the family music environment score (*M* = 3.16, *SD* = 0.52) was moderately high, suggesting that parents in the sample generally provided supportive musical contexts. Children’s empathy scores (*M* = 4.46, *SD* = 0.47) were relatively high with a slight negative skew, consistent with developmental trends in preschool years.

**Table 1:** Descriptive statistics and correlation matrix.

Variable	M	SD	1	2	3	4	5	6	7
1. Child Age	4.45	0.85	1						
2. Gender a	1.47	0.50	0.11	1					
3. Parental Edu	1.81	0.64	0.19	−0.03	1				
4. FME	3.16	0.52	0.17	−0.12	0.20	1			
5. MEC	4.03	0.53	−0.24	−0.02	0.10	0.50**	1		
6. PR	3.41	0.52	−0.05	−0.02	−0.06	0.26**	0.41**	1	
7. Empathy (EC)	4.46	0.47	−0.71	−0.03	0.12	0.36**	0.61**	0.36**	1

*N* = 368. FME, Family Music Environment; MEC, Music Emotional Contagion; PR, Parent–Child Relationship; EC = Empathy. ${\;}^{a}$ Gender: 1 = Boy, 2 = Girl. *p < 0.05, **p < 0.01.

Bold values indicate statistically significant correlations (*p* < 0.05 or *p* < 0.01).

**Figure 1:**
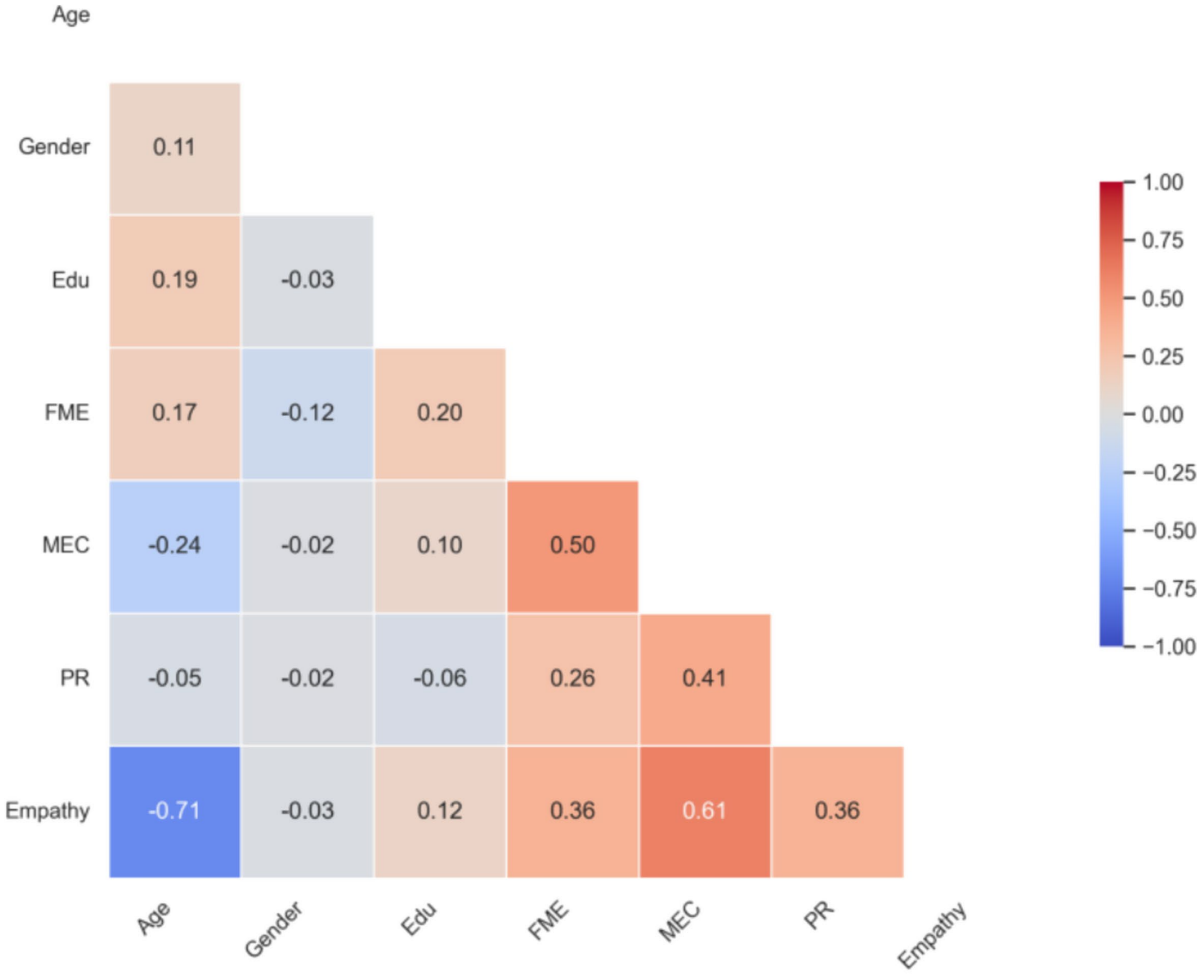
Correlation heatmap of key study variables.

Correlation analyses revealed significant positive associations among the core variables. Figure 1 visually presents the correlation heatmap of these key study variables. As shown in the matrix and the heatmap, the Family Music Environment (FME) was significantly and positively correlated with Children’s Empathy (EC; *r* = 0.36, *p* < 0.01). Furthermore, Music Emotional Contagion (MEC), the proposed mediator, exhibited strong positive correlations with both the independent variable FME (*r* = 0.50, *p* < 0.01) and the dependent variable EC (*r* = 0.61, *p* < 0.01). Additionally, Parent–Child Relationship (PR) quality was positively correlated with all other core variables, with coefficients ranging from 0.26 to 0.41 (*p* < 0.01). These zero-order correlations provide preliminary support for the hypothesized relationships and justify the subsequent testing of the mediation and moderation models.

### 3.3 Mediation analysis of music emotional contagion

To test the mediating role of music emotional contagion, a hierarchical regression analysis was conducted using Hayes’ PROCESS macro (Model 4), controlling for child age, gender, and parental education. The regression results are detailed in Table 2. In the first equation, with Music Emotional Contagion as the dependent variable, the Family Music Environment was found to be a significant positive predictor ( β = 0.445, *t* = 9.89, *p* < 0.001). This indicates that a richer musical environment is associated with higher levels of emotional contagion in children.

**Table 2:** Hierarchical regression analysis results.

Predictor	Dependent variable: MEC		Dependent variable: empathy (EC)	
	β	*t*	β	*t*
Constant		25.71***		8.44***
Age	0.128	4.95***	0.085	3.72***
Gender				
Education	0.024	0.69	0.044	1.45
Family music Env (FME)	0.445	9.89***	0.079	1.83
Music emotional contagion (MEC)			0.418	9.34***
Parent–child relationship (PR)	0.295	6.67***	0.134	3.36***
$R^{2}$	0.376		0.419	
*F*	43.62***			

All coefficients are standardized. ***p < 0.001.

**Figure 2:**
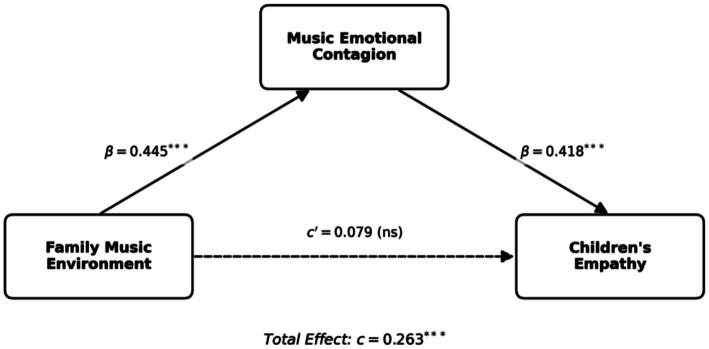
Path diagram of the mediation model.

In the second equation, with Children’s Empathy as the dependent variable, Music Emotional Contagion significantly predicted empathy ( β = 0.418, *t* = 9.34, *p* < 0.001). Notably, when the mediator was included in the model, the direct effect of the Family Music Environment on empathy was no longer statistically significant ( β = 0.079, *t* = 1.83, *p* = 0.068). This pattern suggests that the statistical conditions for mediation are met, supporting the hypothesis that music emotional contagion plays a crucial role in the relationship between the family music environment and children’s empathy. The specific path coefficients and the structure of this mediation model are illustrated in Figure 2.

To robustly verify this mediation effect, a bias-corrected bootstrap analysis with 5,000 resamples was performed. As shown in Table 3, the indirect effect of FME on EC via MEC was estimated at 0.185, with a 95% confidence interval (CI) of [0.138, 0.239]. Since the confidence interval does not encompass zero, the mediation effect is statistically significant. In contrast, the direct effect was 0.078 with a 95% CI of [−0.002, 0.153], which includes zero. These findings support a full mediation model statistically, indicating that the association between the family music environment and children’s empathy is largely explained by the mechanism of music emotional contagion.

**Table 3:** Bootstrap analysis of mediation effects.

Path	Effect	Boot SE	Lower 95% CI	Upper 95% CI	Conclusion
Indirect effect (FME → MEC → EC)	0.185	0.026	0.138	0.239	Significant
Direct effect (FME → EC)	0.078	0.039	−0.002	0.153	Not Sig.
Total effect (FME → EC)	0.263	0.040	0.184	0.342	Significant

### 3.4 Moderated mediation analysis

Finally, PROCESS Model 7 was utilized to examine whether Parent–Child Relationship (PR) quality moderated the first stage of the mediation process (FME → MEC). The analysis revealed that the interaction term (FME$\timesPR) was not statistically significant ( \beta$ = 0.070, *t* = 0.92, *p* = 0.357). This result indicates that the strength of the relationship between the family music environment and children’s music emotional contagion does not significantly vary across different levels of parent–child relationship quality.

**Figure 3:**
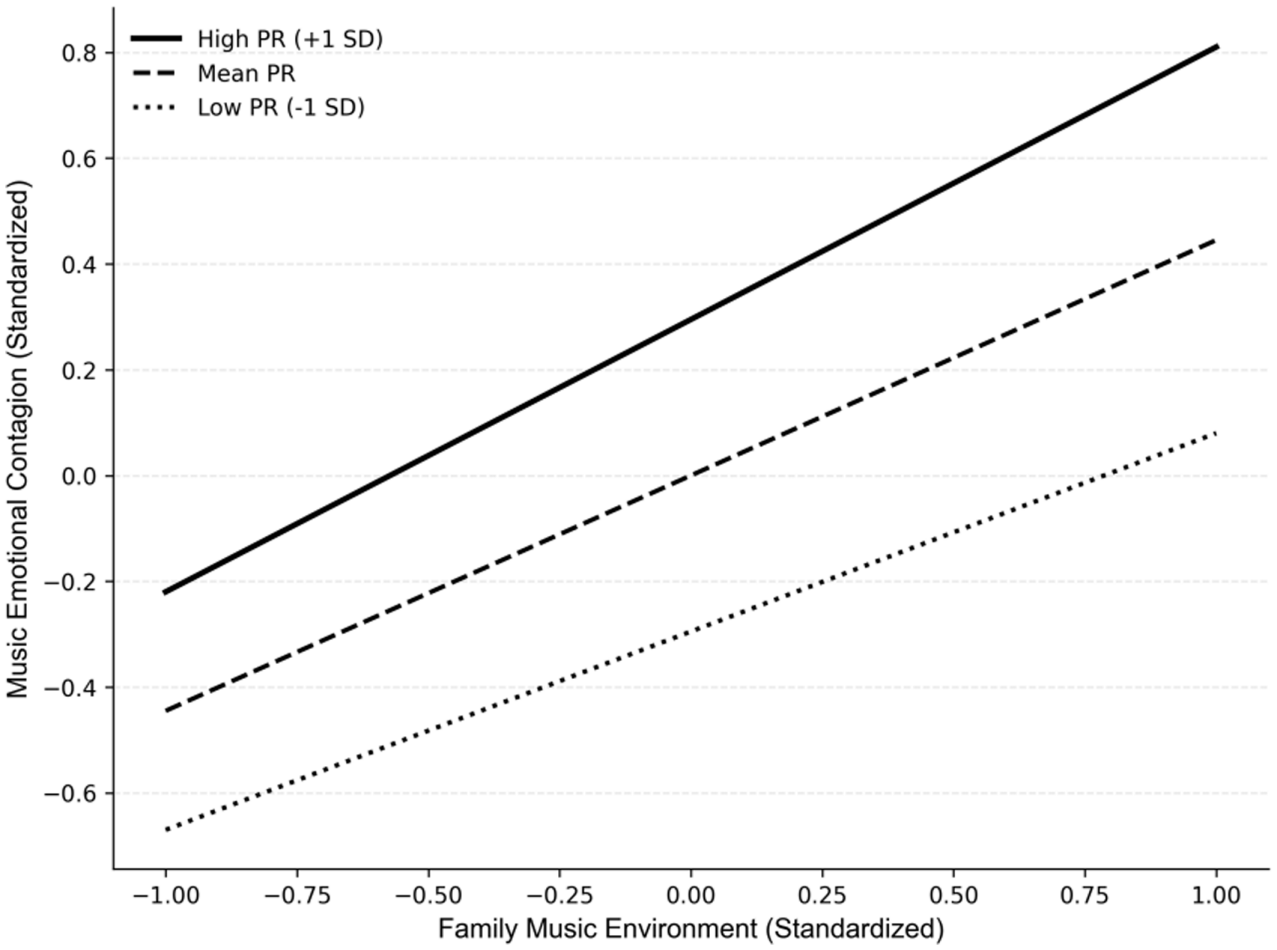
Simple slope analysis plot. Although the interaction effect was not statistically significant (*p* > 0.05), this figure is included to illustrate the parallel positive contributions of both family music environment and parent–child relationship.

Despite the non-significant interaction, the parent–child relationship exerted a significant main effect on music emotional contagion ( β = 0.295, *p* < 0.001). Figure 3 is presented to illustrate this pattern: while a high-quality parent–child relationship independently contributes to higher levels of emotional contagion, the slopes representing the effect of the music environment remain parallel, suggesting that the music environment’s effect is consistent regardless of relationship quality. The analysis of conditional indirect effects, presented in Table 4, further confirmed this pattern. The indirect effect of the family music environment on empathy through music emotional contagion remained significant and numerically stable across low (−1 *SD*), mean, and high (+1 *SD*) levels of parent–child relationship quality, with substantially overlapping confidence intervals.

**Table 4:** Conditional indirect effects at different levels of parent–child relationship.

Level of PR	Indirect effect	Boot SE	Lower 95% CI	Upper 95% CI
Low (−1 *SD*)	0.171	0.040	0.092	0.249
Mean	0.186	0.040	0.108	0.264
High (+1 *SD*)	0.201	0.040	0.123	0.280

PR, Parent–Child Relationship. The interaction effect was non-significant (**p > 0.05), indicating that the mediation mechanism is robust across varying levels of relationship quality**.

## 4 Discussion

Grounded in an integrated theoretical framework encompassing Ecological Systems Theory, Emotional Contagion Theory, and Attachment Theory, the present study systematically examined the mechanisms and boundary conditions through which the family music environment is associated with young children’s empathy. By constructing a moderated mediation model, we provide empirical support for understanding how external environmental factors may be translated into internal socio-emotional competencies. The findings reveal a theoretically compelling pattern: the association between the family music environment and children’s empathy is statistically fully mediated by music emotional contagion. Notably, this indirect pathway demonstrates stability and universality across different levels of parent–child relationship quality. These results not only clarify the psychological “process” linking home context to social competence but also offer a nuanced perspective on the traditional “relationship-centered” view in family education, suggesting that music-related resources might function as stable protective factors.

### 4.1 Opening the “black box”: the psychologicalization of environmental influence

A primary contribution of this study is the identification of the statistical full mediation effect of music emotional contagion. After introducing the mediator, the direct predictive effect of the family music environment on empathy became non-significant. This statistical transition carries profound theoretical implications: it suggests that the family music environment—in its physical sense, such as the mere availability of instruments or the frequency of background music—may not automatically generate empathetic capacity. Instead, its influence appears to undergo a process of “psychologicalization,” whereby the external environment successfully activates children’s internal mechanisms of emotional sensitivity and resonance.

This finding aligns with and extends the “Embodied Simulation” hypothesis in music psychology (Koelsch, 2014). Music acts as a high-fidelity “affective simulator.” Its structural elements—tempo, dynamics, timbre, and contour—mirror the prosodic and kinematic features of human emotional expression (Juslin and Laukka, 2003). When children engage in musical activities within the family, they are not merely passive listeners; they are effectively practicing “emotional gymnastics” in a safe, non-threatening context. The repeated activation of perceptual-motor neural circuits during musical experiences creates and refines an “emotional mirror system” or Mirror Neuron System (MNS; Cirelli et al., 2014; Savage et al., 2020).

Furthermore, this study elucidates why the family context is critical for this mechanism. Unlike solitary listening, the family music environment often involves joint attention and interpersonal synchrony. This shared experience likely amplifies the contagion effect. As children perceive the emotional content of music, they simultaneously observe their parents’ emotional congruency. This dual input reinforces the link between auditory cues and internal affective states, scaffolding the child’s ability to decode emotional signals. Consequently, this music-trained sensitivity can be recruited during social interactions, allowing children to more effectively simulate and resonate with the emotional states of peers, thereby facilitating empathy.

### 4.2 “Parallel Positive Effects” in the family system

One of the most thought-provoking findings—which refines conventional assumptions in developmental psychology—is that parent–child relationship quality did not moderate the effect of the family music environment (interaction *p* > 0.05). While high-quality parent–child relationships significantly predicted higher levels of emotional contagion (main effect), the slope of the relationship between the music environment and emotional contagion remained consistent across different levels of attachment security.

Theoretically, this suggests a “Parallel Positive Effects” characteristic of family subsystems, where the “cultural-esthetic subsystem” (music environment) and the “relational-attachment subsystem” (parent–child bond) may operate through distinct pathways. Traditional attachment perspectives often imply a “gating model,” assuming that educational inputs are only effective when embedded within a secure, warm relational context. However, our results do not support this interactionist assumption in the context of music. The mechanism through which music operates appears relatively independent of the attachment system’s regulatory function. This independence likely stems from the non-verbal and non-evaluative nature of music (Cross, 2003). Unlike verbal instruction, which relies heavily on parental authority, musical experience directly stimulates the auditory and limbic systems without necessarily requiring complex cognitive appraisal of the relationship.

Based on the significant main effect of the parent–child relationship combined with the non-significant interaction, we tentatively propose a “Resource Compensation Possibility.” While high-quality parent–child relationships indeed elevate children’s baseline emotional development, the incremental benefit of the music environment remains effective even in families with less optimal relationship quality. This implies that the family music environment could serve as a compensatory protective factor. For families experiencing relational strain, or for parents who are less verbally expressive, enriching the home musical environment might offer an alternative channel for emotional nourishment. It allows children to potentially maintain a trajectory of empathy development even when the relational climate is not ideal.

### 4.3 Practical implications: from “relationship repair” to “environmental intervention”

The findings of this study have significant implications for parenting practices and early childhood education.

For Parents: The study suggests a paradigm shift from “waiting for relationship repair” to “active environmental intervention.” Many parents feel paralyzed by the belief that they must first establish a perfect attachment before their educational efforts can bear fruit. Our findings offer a more encouraging message: Start with music now. Even if the parent–child relationship is currently tense, introducing a rich music environment can still effectively support the child’s emotional sensitivity. Music can serve as a non-intrusive “emotional lubricant” or regulation tool (Saarikallio, 2010), fostering a shared emotional atmosphere without the pressure of direct verbal interaction.

For Educators: Early childhood educators should recognize that empathy training need not rely exclusively on verbal reasoning. “Implicit emotional training” through music is highly effective. Curricula should integrate activities that focus on “listening and feeling,” using music to induce different moods and guide children to experience these shifts, thereby training the neural substrates of empathy in a direct, embodied manner.

### 4.4 Limitations and future directions

Despite its contributions, several limitations warrant careful consideration.

First, regarding Common Method Bias (CMB), Harman’s single-factor test showed that the first factor accounted for 56.78% of the variance, which exceeds the conventional threshold of 40%. Although CFA results supported the discriminant validity of the constructs, we explicitly acknowledge that the reliance on parent-reported data for both the family environment and children’s empathy constitutes a significant limitation. This shared source variance may have systematically inflated the observed correlations and the mediation effect size. Therefore, the strength of the relationships reported here should be interpreted with caution. Future studies must employ multi-informant approaches (e.g., combining teacher ratings, peer assessments, or observational data) to mitigate this bias and verify the robustness of the findings.

Second, the cross-sectional design of this study strictly limits our ability to make causal inferences. While the moderated mediation model provides a theoretical explanation for the relationships (“environment → contagion → empathy”), we cannot rule out bidirectional effects. For instance, children with higher innate empathy might elicit more musical interaction from their parents. The term “mechanism” used in this study refers to statistical mediation within the theoretical framework, not a confirmed longitudinal trajectory. Future longitudinal tracking or experimental intervention studies are necessary to confirm the directionality and developmental sequence of these effects.

Third, the measurement of “music emotional contagion” relied on situational experiments with self-reports. Future research could integrate physiological measures (e.g., skin conductance, heart rate variability) to objectively capture the activation of the mirror neuron system. Finally, the study treated “family music environment” as a unitary construct. Future research should differentiate between participation types (active music-making vs. passive listening) to determine if specific types of musical engagement yield differential effects.

## 5 Conclusion

The family music environment is positively associated with young children’s empathy. Statistical analysis suggests that this association is mediated by music emotional contagion, which may work by “tuning” the child’s emotional sensitivity. Crucially, the parent–child relationship does not moderate the strength of this mediation path; instead, the music environment and parent–child relationship appear to operate as parallel positive forces. This suggests that the family music environment could function as a robust educational resource with the potential to promote empathy across varying levels of attachment security, thereby providing a valuable compensatory strategy for fostering socio-emotional development.

## Data Availability

The raw data supporting the conclusions of this article will be made available by the authors, without undue reservation.
